# The Dynamic Coenzyme Network of B Vitamins in Nutritional Neuropathy and Neuropsychiatric Vulnerability: A Mechanistic Narrative Review

**DOI:** 10.3390/nu18142306

**Published:** 2026-07-14

**Authors:** Yonghyun Yoon, Jihyo Hwang, Rowook Park, Jaehyun Shim, King Hei Stanley Lam, Jeimylo C. de Castro, Teinny Suryadi, Anwar Suhaimi, Chan-Mo Yang

**Affiliations:** 1 Department of Orthopaedic Surgery, Gangnam Sacred Heart Hospital, Hallym University College of Medicine, 1 Singil-ro, Yeongdeungpo-gu, Seoul 07441, Republic of Korea; mgyyh00@gmail.com (Y.Y.); hwangjihyo36@gmail.com (J.H.); 2 Incheon Terminal Orthopedic Surgery Clinic, Inha-ro 489beon-gil, Namdong-gu, Incheon 21574, Republic of Korea; 3 International Academy of Regenerative Medicine, Inha-ro 489beon-gil, Namdong-gu, Incheon 21574, Republic of Korea; jhyunshim@gmail.com; 4 The Board of Clinical Research, The Hong Kong Institute of Musculoskeletal Medicine, Kowloon, Hong Kong; painfreedoc22@gmail.com (T.S.); anwar@ummc.edu.my (A.S.); 5 Department of Rehabilitation Medicine, Sae Yonsei Rehabilitation Clinic, Seoul 03186, Republic of Korea; 6 Department of Neurosurgery, Chungdammadi Neurosurgery Clinic, Seoul 03186, Republic of Korea; 7 SMARTMD Center for Non-Surgical Pain Interventions, Makati 1205, Philippines; jeidec@yahoo.com.ph; 8 Department of Psychiatry, School of Medicine, Wonkwang University, Iksan 54649, Republic of Korea; ychanmo@wku.ac.kr

**Keywords:** B vitamins, nutritional neuropathy, mitochondrial metabolism, coenzyme network, functional deficiency, one-carbon metabolism, homocysteine, cobalamin, folate, medication-associated deficiency, neuropsychiatric vulnerability, narrative review

## Abstract

Nutritional peripheral neuropathy is a clinically important condition associated with inadequate intake, malabsorption, bariatric surgery, aging, metabolic disease, and chronic medication exposure. B vitamins serve as essential coenzymes in mitochondrial energy metabolism, one-carbon metabolism, neurotransmitter synthesis, redox regulation, and myelin maintenance. This narrative review synthesizes mechanistic and clinical evidence linking B vitamin-dependent coenzyme systems to nutritional neuropathy and selected neuropsychiatric manifestations. Particular emphasis is placed on the tricarboxylic acid cycle, electron transport chain, urea cycle, folate–methionine cycle, and MTHFR-dependent one-carbon metabolism. The proposed “dynamic coenzyme network” is intended as an integrative mechanistic framework rather than a stand-alone therapeutic recommendation. Although biochemical interdependence among B vitamins supports evaluation for combined or functional deficiencies in selected patients, mechanistic plausibility should not be equated with clinical efficacy. Clinical consideration of B vitamin repletion should be guided by documented deficiency, dietary and gastrointestinal risk factors, medication exposure, neurological phenotype, and functional biomarkers such as homocysteine and methylmalonic acid. Adjunctive nutrients, including alpha-lipoic acid and acetyl-L-carnitine, may have context-specific relevance, particularly in metabolic neuropathies, but evidence for fixed multi-nutrient combinations remains heterogeneous. Further well-designed clinical studies are needed to determine whether this mechanistic framework translates into meaningful neurological or neuropsychiatric benefit beyond established deficiency states.

## 1. Introduction

Peripheral neuropathy arising from nutritional deficiency is a global problem influenced by dietary restriction, gastrointestinal disease, bariatric surgery, aging, socioeconomic factors, and medication-related impairment of nutrient absorption or metabolism [1,2,3]. Among acquired neuropathies, those secondary to nutritional deficiencies are particularly important because many can be stabilized or reversed with adequate, timely repletion and supportive care [4]. The vitamins most vital for optimal functioning of the peripheral nerves include the B vitamins—B1/thiamine, B2/riboflavin, B3/niacin, B5/pantothenic acid, B6/pyridoxine, B9/folate, and B12/cobalamin—each fulfilling unique but deeply interconnected biochemical roles [5].

Despite this complexity, clinical and scientific attention has historically focused on only a small subset of B vitamins—most commonly B1, B6, and B12—while the contributions of riboflavin, niacin, pantothenic acid, and folate to neurological health have received comparatively little attention [6]. Furthermore, supplementation of a single deficient vitamin in isolation may fail to account for metabolic interdependencies among B vitamins [5,7]. Basic and clinical evidence suggests that B vitamins have non-substitutable biochemical roles, although the clinical implications of this interdependence vary by deficiency state, disease context, and available evidence [5].

The neurological consequences of B vitamin insufficiency should not be interpreted only within the narrow framework of peripheral axonal degeneration. Peripheral nerves and the central nervous system share a dependence on mitochondrial energy metabolism, one-carbon methylation, neurotransmitter synthesis, redox balance, and myelin maintenance. Accordingly, B vitamin deficiency has been associated with a spectrum of neurological and neuropsychiatric manifestations, although the strength of evidence and causal certainty differ substantially across conditions [6,8,9]. This peripheral–central continuum is particularly relevant for cobalamin and folate deficiency, in which elevated methylmalonic acid and homocysteine, impaired methionine synthase activity, reduced S-adenosylmethionine availability, and altered methylation capacity may affect both myelin integrity and neurotransmitter metabolism [8,10,11,12].

A further clinically important but often underrecognized issue is that B vitamin deficiency is not always caused by insufficient dietary intake alone. Chronic medication exposure may reduce B vitamin availability through impaired absorption, altered transport, enzyme inhibition, accelerated catabolism, increased urinary loss, or antimetabolite effects. Representative examples include metformin-associated vitamin B12 deficiency, gastric acid suppression-associated impairment of food-bound cobalamin absorption, isoniazid-associated pyridoxine deficiency, antifolate effects of methotrexate, antiepileptic drug-associated reductions in folate or pyridoxal 5′-phosphate, levodopa-associated alterations in homocysteine and B vitamin metabolism, and loop diuretic-associated thiamine loss [3,13]. These mechanisms are particularly relevant in patients with neuropathy, diabetes, aging-related disorders, psychiatric disease, chronic pain, or polypharmacy, in whom nutritional risk may be compounded by metabolic and pharmacological stressors.

The purpose of this review is to synthesize established biochemical pathways and clinically relevant evidence regarding B vitamin-dependent coenzyme systems in nutritional neuropathy and neuropsychiatric vulnerability. The proposed “dynamic coenzyme network” does not represent a new metabolic pathway; rather, it is an integrative framework for interpreting how established vitamin-dependent pathways may converge in nutritionally or pharmacologically vulnerable patients. We emphasize the distinction between mechanistic plausibility, preclinical evidence, observational associations, and interventional clinical data. Accordingly, this review discusses B vitamin repletion as a context-specific clinical consideration guided by documented deficiency, risk factors, medication exposure, and functional biomarkers, rather than as a universal recommendation for broad supplementation.

## 2. Methods

This narrative review was designed to synthesize mechanistic and clinical evidence regarding B vitamin-dependent coenzyme networks in nutritional neuropathy and neuropsychiatric vulnerability. A literature search was performed in PubMed/MEDLINE, Scopus, and Google Scholar from database inception to April 2026. Search terms included combinations of “B vitamins,” “thiamine,” “riboflavin,” “niacin,” “pantothenic acid,” “pyridoxine,” “folate,” “cobalamin,” “nutritional neuropathy,” “peripheral neuropathy,” “mitochondrial metabolism,” “TCA cycle,” “one-carbon metabolism,” “homocysteine,” “MTHFR,” “neuropsychiatric symptoms,” “metformin,” “proton pump inhibitors,” “antiepileptic drugs,” “levodopa,” “alpha-lipoic acid,” and “acetyl-L-carnitine.”

We prioritized peer-reviewed reviews, clinical practice guidelines, randomized controlled trials, systematic reviews, meta-analyses, and mechanistic studies directly relevant to B vitamin metabolism and neural function. Preclinical studies were included when they clarified biochemical mechanisms but were not interpreted as direct clinical evidence. Articles were excluded if they were unrelated to neural metabolism, focused exclusively on non-neurological outcomes, or did not provide sufficient mechanistic or clinical relevance. Because this was a narrative review rather than a systematic review, formal risk-of-bias assessment and meta-analysis were not performed.

## 3. Overview of Nutritional Neuropathy

Nutritional deficiencies may result from reduced intake—whether from starvation, foods poor in nutritional content, or restricted diets—or from reduced absorption due to gastrointestinal disease, prior bariatric surgery, or autoimmune conditions such as pernicious anemia [14,15,16]. Importantly, nutritional neuropathies due to poor intake or malabsorption frequently result from several concomitant deficiencies rather than a single isolated deficiency [4,17]. Although these neuropathies assume different clinical patterns, most are length-dependent, sensory axonopathies; cobalamin deficiency neuropathy is the exception, often presenting with a length-dependent sensory neuropathy, frequently accompanied by subacute combined degeneration of the spinal cord.

Mitochondrial dysfunction is one important mechanism through which nutritional deficiencies may affect neural tissue, particularly because neurons have high energy requirements. However, nutritional neuropathies are mechanistically heterogeneous and may also involve impaired myelin maintenance, altered one-carbon metabolism, oxidative stress, axonal degeneration, and medication- or disease-related metabolic stress. In neuronal tissue especially, damage to mitochondria due to the lack of crucial nutrients—such as B vitamins required for correct mitochondrial function—causes an interruption of electron transport and a consequent reduction in ATP production, leading to impaired cellular vitality and ultimately to cell death [18,19]. Thiamine deficiency leads to selective neuronal cell death by multiple mechanisms: cellular energy failure, focal lactic acidosis, blood–brain barrier breakdown, mitochondrial dysfunction, and oxidative stress [20,21]. Beyond energy failure, other mechanisms include axon demyelination, particularly for B12 and folate deficiencies, and oxidative stress resulting from the loss of antioxidant functions of these nutrients [10,11].

## 4. B Vitamin Coenzymes in Mitochondrial Energy Metabolism

The tricarboxylic acid (TCA) cycle—also known as the Krebs cycle or citric acid cycle—is a series of chemical reactions in the mitochondria that generate energy in the form of ATP from carbohydrates, fats, and proteins. Carbohydrates, fats, and proteins are first converted to acetyl-CoA, most often via pyruvate, and then undergo eight enzymatic reactions that result in the production of NADH and FADH2, which transfer the energy generated by the TCA cycle to the electron transport chain, ultimately leading to ATP synthesis. Because neurons have high energy requirements and limited capacity to store high-energy compounds, sustained ATP production is important for neuronal integrity [22].

Of critical significance, the active coenzyme forms derived from B1/thiamine, B2/riboflavin, B3/niacin, and B5/pantothenic acid are essential coenzymes in mitochondrial aerobic respiration and cellular energy production via their direct roles in the citric acid cycle, the electron transport chain, and the resultant formation of ATP. Acetyl-CoA, which incorporates B5/pantothenic acid-derived coenzyme A, provides the main substrate for the TCA cycle. In addition, B1/thiamine and B12/cobalamin play distinct, intersecting roles in the mitochondrial metabolism of glucose and fatty acids and amino acids, respectively, thereby contributing substrates to the citric acid cycle. B6/PLP, B9/folate, and B12/cobalamin further participate through anaplerotic and one-carbon-related pathways that replenish TCA cycle intermediates, and through the methionine cycle whose products are essential for myelin maintenance. These relationships provide a biochemical basis for considering B vitamin status as an interconnected system, although the clinical relevance of each vitamin depends on the specific deficiency state, disease context, and level of supporting evidence.

### 4.1. B Vitamins as Coenzymes: Catalytic Recycling, Not Substrate Consumption

**Figure 1:**
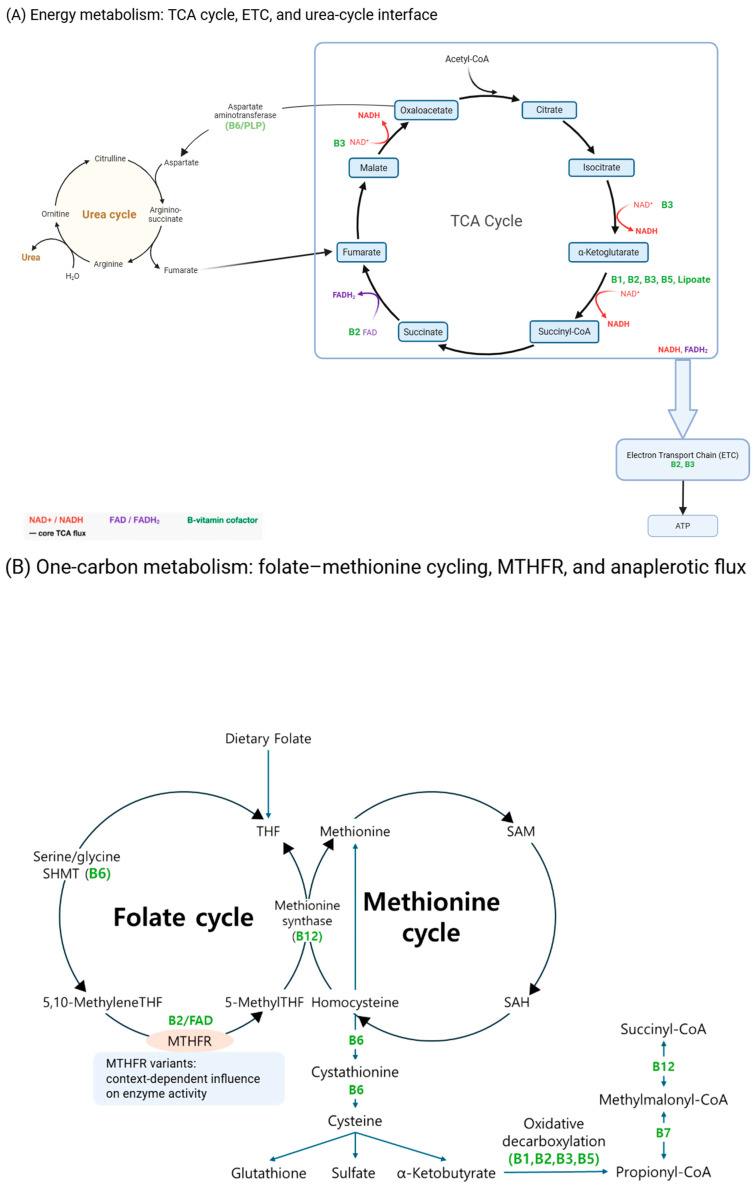
B vitamin-dependent coenzyme networks linking energy metabolism, one-carbon metabolism, and neural vulnerability. (**A**) Energy metabolism: TCA cycle, ETC, and urea-cycle interface. The tricarboxylic acid (TCA) cycle is shown as a central mitochondrial hub that generates reducing equivalents for the electron transport chain (ETC). B3/niacin-derived NAD^+^/NADH and B2/riboflavin-derived FAD/FADH_2_ participate in TCA-cycle dehydrogenase reactions and electron transfer to the ETC. B1/thiamine, B2/riboflavin, B3/niacin, B5/pantothenic acid, and lipoate support the α-ketoglutarate dehydrogenase complex. Although not separately depicted, acetyl-CoA generation from pyruvate also depends on the pyruvate dehydrogenase complex, which requires B1, B2, B3, B5, and lipoate. Pyridoxal 5′-phosphate (B6/PLP) links oxaloacetate and aspartate metabolism through aminotransferase activity, thereby connecting the TCA cycle with the urea cycle. (**B**) One-carbon metabolism: folate–methionine cycling, MTHFR, and anaplerotic flux. Riboflavin-derived FAD supports methylenetetrahydrofolate reductase (MTHFR) activity, folate provides one-carbon units, B12/cobalamin supports methionine synthase, and B6/PLP contributes to serine–glycine metabolism and transsulfuration. Homocysteine metabolism intersects with methylation capacity through methionine, S-adenosylmethionine (SAM), and S-adenosylhomocysteine (SAH), and with TCA-related anaplerosis through α-ketobutyrate, propionyl-CoA, methylmalonyl-CoA, and succinyl-CoA. The transsulfuration pathway is simplified for schematic clarity; cystathionine-derived products include cysteine and α-ketobutyrate. MTHFR variants may influence enzyme activity in a context-dependent manner, but genotype alone should not be interpreted as a stand-alone indication for supplementation. Arrows schematically indicate the direction of metabolic reactions or pathway connections rather than quantitative flux. In panel A, black arrows denote core TCA-cycle flux, red and purple curved arrows denote NAD^+^/NADH and FAD/FADH2 redox cycling, respectively, and gray or light-blue arrows indicate coupling with the urea cycle and the ETC. In panel B, black arrows denote the principal folate–methionine cycle reactions, whereas blue arrows highlight selected pathway inputs, cross-cycle connections, and downstream or anaplerotic branches. Abbreviations: ETC, electron transport chain; FAD, flavin adenine dinucleotide; FADH_2_, reduced flavin adenine dinucleotide; MTHFR, methylenetetrahydrofolate reductase; NAD^+^, nicotinamide adenine dinucleotide; NADH, reduced nicotinamide adenine dinucleotide; PLP, pyridoxal 5′-phosphate; SAH, S-adenosylhomocysteine; SAM, S-adenosylmethionine; TCA, tricarboxylic acid; THF, tetrahydrofolate.

A foundational concept essential to understanding B vitamin physiology is that these vitamins function as coenzymes (catalysts), not substrates (Figure 1)—they are neither consumed nor destroyed during the enzymatic reactions they facilitate. Instead, they are chemically transformed into a transiently modified form and then quantitatively regenerated, enabling each molecule to participate in thousands of reaction cycles. This catalytic economy has profound implications: even modest reductions in coenzyme availability may influence multiple enzyme systems, particularly in tissues with high metabolic demand, and a shift in the redox state of the mitochondria (due, for example, to pathological electron transport chain dysfunction) can secondarily inactivate B vitamin coenzymes [18,23].

Within the TCA cycle, the key recycling mechanisms operate as follows. NAD^+^, derived from B3/niacin, accepts a hydride ion (H^−^) from TCA substrates at three dehydrogenase reactions—isocitrate dehydrogenase, α-ketoglutarate dehydrogenase, and malate dehydrogenase—becoming NADH. NADH is not stored but immediately donates its electrons to Complex I of the electron transport chain (ETC), regenerating NAD^+^. This regeneration is functionally important: if the ETC is impaired, NADH may accumulate and NAD^+^ availability may decline, thereby limiting NAD^+^-dependent TCA dehydrogenase reactions, even when B3 levels are otherwise adequate [24,25]. FAD, derived from B2/riboflavin, undergoes an analogous cycle: it accepts electrons from succinate at succinate dehydrogenase (Complex II), becoming FADH_2_, and is regenerated by donating electrons to ubiquinone within the ETC. The entire FAD pool is continuously cycled in this manner. Critically, FAD is also the essential cofactor for MTHFR; if the FAD pool is diminished, the MTHFR reaction is impaired, reducing the regeneration of methionine from homocysteine [26].

Coenzyme A (CoA), derived from B5/pantothenic acid, operates by a carrier (thioester) mechanism: CoA transiently binds acetyl or acyl groups as acetyl-CoA or succinyl-CoA and is released in free form at the citrate synthase reaction and at the succinyl-CoA synthetase reaction. The released free CoA is immediately available for reuse—CoA itself is neither consumed nor degraded in these reactions. Thiamine pyrophosphate (TPP), derived from B1/thiamine, functions as a cofactor of the PDH and α-KGDH multienzyme complexes: it transiently accepts an acyl group from the decarboxylation product and transfers it to the lipoic acid moiety on the E2 subunit, then is regenerated in free form, ready for the next catalytic cycle [22]. Pyridoxal 5′-phosphate (PLP), derived from B6/pyridoxine, forms a Schiff-base intermediate with the amino group of the substrate, and after the transamination reaction, the pyridoxamine 5′-phosphate (PMP) form is regenerated back to PLP by the second half-reaction with α-ketoacids—completing a full catalytic cycle.

### 4.2. Functional Coenzyme Availability Despite Catalytic Recycling

Although B vitamins function as catalytic coenzymes that are regenerated during enzymatic reactions, this biochemical recycling does not imply that the total body pool of coenzymes is static or inexhaustible. Rather, multiple physiological and pathological processes may deplete or limit the availability of functional coenzyme pools, supporting the need for adequate dietary intake and, when clinically indicated, targeted repletion [27,28].

First, unlike fat-soluble vitamins, B vitamins are water-soluble and are not stored in significant quantities in the body. They are continuously filtered and excreted via the kidneys, resulting in a steady loss of total molecular pool. The concept of “recycling” applies only to the intra-cycle chemical regeneration of coenzymes within enzymatic reactions, not to the preservation of the total systemic pool. Consequently, adequate nutritional intake remains necessary to maintain systemic vitamin availability, particularly in patients with reduced intake, malabsorption, renal loss, or increased metabolic demand [29].

Second, coenzyme molecules themselves are subject to biological turnover. Cellular and protein turnover requires constant resynthesis of apoenzymes, and many vitamin-dependent enzymes must bind their vitamin-derived cofactors to achieve or maintain catalytically competent conformations [30,31]. Even in non-dividing cells such as neurons, continuous turnover of axonal proteins, synaptic components, and mitochondrial enzymes imposes a sustained demand for newly available coenzymes [32,33]. Given the exceptionally high metabolic activity of neuronal tissue, this requirement is particularly pronounced in the nervous system.

Third, functional deficiency may arise independently of total vitamin levels due to metabolic trapping. As discussed in Section 4.1, impairment of the electron transport chain leads to accumulation of NADH and depletion of NAD^+^, effectively reducing the pool of functionally available coenzyme despite adequate B3/niacin status [34,35]. This phenomenon is particularly relevant in metabolic disorders such as diabetes and aging-related conditions, where mitochondrial dysfunction and redox imbalance are well established contributors to neuropathy. In such cases, restoration of function may require both correction of the underlying pathology and increased substrate availability [36].

Fourth, coenzyme molecules may undergo irreversible oxidative or structural damage—such as irreversible overoxidation of lipoamide or depletion of NAD^+^ pools—under conditions of oxidative stress, which is a hallmark of neuropathic and metabolic disorders. Damaged coenzyme-related molecules may be removed from catalytic cycles and require replacement through ongoing cellular turnover and vitamin availability, further increasing demand [37,38].

Finally, disease states such as inflammation, neuropathy, and metabolic disorders are characterized by simultaneous increases in energy demand and oxidative stress. These conditions accelerate coenzyme turnover and impair recycling efficiency, ultimately exceeding the capacity of endogenous systems constrained by substrate availability, enzymatic capacity, and redox imbalance. As a result, endogenous regeneration may become insufficient in selected high-risk states, in which assessment of vitamin status and clinically guided repletion may be appropriate [39,40,41].

Taken together, these mechanisms indicate that while B vitamins are not consumed in individual enzymatic reactions, the total functional pool of coenzymes is dynamic and sensitive to physiological and pathological conditions. Catalytic recycling therefore does not eliminate the need for adequate nutritional intake. Thus, the apparent paradox of catalytic recycling and clinical vulnerability can be understood by recognizing that coenzyme systems operate within an open, dynamic biological network subject to loss, damage, and functional inactivation. This concept provides a functional, systems-based perspective of coenzyme availability that complements the traditional view of vitamin sufficiency based primarily on circulating levels.

## 5. Individual B Vitamins: TCA Cycle Roles and Neuropathological Consequences of Deficiency

### 5.1. B1/Thiamine

Thiamine is arguably the B vitamin with the most direct and well-established connection to both the TCA cycle and peripheral neuropathy. After phosphorylation within cells, free thiamine is converted to its biologically active form, thiamine pyrophosphate (TPP), which acts as a coenzyme for three major enzymes in glucose metabolism: transketolase (TK) in the pentose phosphate pathway, pyruvate dehydrogenase (PDH) in the pyruvate-to-acetyl-CoA conversion linking glycolysis to the TCA cycle, and alpha-ketoglutarate dehydrogenase (α-KGDH) within the TCA cycle itself [26]. PDH enables the formation of acetyl-CoA—the primary entry point of substrates into the TCA cycle—and also serves as a precursor for the neurotransmitter acetylcholine and for myelin synthesis. α-KGDH catalyzes the conversion of α-ketoglutarate to succinyl-CoA within the TCA cycle, thereby sustaining cycle flux and regulating the levels of neurotransmitters including glutamate, GABA, and aspartate.

In mammalian tissue, thiamine pyrophosphate (TPP) functions as an essential coenzyme for key multienzyme complexes, primarily including transketolase (important in maintenance of the myelin sheath), pyruvate dehydrogenase (energy production in the TCA cycle), α-ketoglutarate dehydrogenase (neurotransmitter synthesis), and branched-chain keto acid dehydrogenase. TK activity is the most sensitive to variations in thiamine concentrations in brain regions; consequently, thiamine deficiency leads to increased oxidative stress, reduced cell proliferation, and decreased synthesis of fatty acids including myelin [1]. Clinically, long-term thiamine deficiency is associated with dry beriberi—a length-dependent large fiber sensory and motor axonal neuropathy—and Wernicke–Korsakoff syndrome at the central level [5,42].

### 5.2. B2/Riboflavin

Riboflavin plays a pivotal role in the TCA cycle and mitochondrial electron transport chain through its biologically active coenzyme forms: flavin mononucleotide (FMN) and flavin adenine dinucleotide (FAD). These flavocoenzymes ensure the functionality of numerous flavoproteins including dehydrogenases, oxidases, and reductases, which play pivotal roles in the mitochondrial electron transport chain, β-oxidation of fatty acids, redox homeostasis, and the citric acid cycle itself. Riboflavin-derived FAD serves as a direct cofactor for succinate dehydrogenase (Complex II of the ETC), which catalyzes the conversion of succinate to fumarate within the TCA cycle while simultaneously feeding electrons into the respiratory chain.

Beyond its direct TCA cycle role, riboflavin is uniquely positioned as a regulator of other B vitamins: the flavoproteins are involved in the synthesis, conversion, and recycling of B3/niacin, B9/folate, and B6-related coenzymes, and for the synthesis of all heme proteins [43]. B2/riboflavin is also an obligate coenzyme with MTHFR in the folate cycle, and rate-limits the recycling of methionine synthase in the methionine cycle [43,44,45]. The conversion of pyridoxine to its active form PLP relies on pyridoxine 5′-phosphate oxidase, a flavin mononucleotide-dependent enzyme; thus, B2/riboflavin deficiency can indirectly impair B6/PLP-dependent function. FAD-dependent processes may also influence cobalamin-related metabolism, although the clinical relevance of this interaction requires context-specific interpretation [5].

### 5.3. B3/Niacin

B3/niacin is converted to the coenzymes NAD^+^ and NADP^+^, which participate in oxidative reactions important for energy production. Within the TCA cycle, NAD^+^ functions as the electron acceptor for three critical dehydrogenase reactions—isocitrate dehydrogenase, α-ketoglutarate dehydrogenase, and malate dehydrogenase—making it indispensable for cycle flux and NADH generation that drives ATP synthesis through the ETC. A vast array of processes and enzymes involved in every aspect of peripheral and brain cell function depend on niacin-derived nucleotides, including oxidative reactions, antioxidant protection, DNA metabolism and repair, and cellular signaling events [46,47,48].

B3/niacin deficiency may reduce the NAD^+^/NADH ratio, impaired TCA cycle function, and promotion of anaerobic glycolysis at the expense of mitochondrial oxidative phosphorylation [47,48]. Clinically, pellagra—the syndrome of niacin deficiency—is characterized by dermatitis, diarrhea, and dementia, the last reflecting the profound dependence of the nervous system on adequate NAD^+^ availability [49,50].

### 5.4. B5/Pantothenic Acid

B5/pantothenic acid is the obligatory precursor to coenzyme A (CoA), which is utilized as a fundamental coenzyme in numerous metabolic processes—most notably in the pyruvate oxidation step entering the TCA cycle and during both synthesis and oxidation of fatty acids. Acetyl-CoA, formed from pantothenate-derived CoA, is the principal substrate entering the TCA cycle through condensation with oxaloacetate to form citrate. The pantothenate-derived CoA is also required by the PDH complex and succinyl-CoA synthetase within the TCA cycle, making B5 an essential structural component of the cycle itself.

B5/pantothenic acid deficiency may impair brain energy metabolism by impairing the levels of four mitochondrial TCA-cycle enzymes: the pyruvate dehydrogenase complex, isocitrate dehydrogenase, the α-ketoglutarate dehydrogenase complex, and succinyl-CoA synthetase—all of whose functions are modulated directly or indirectly by CoA [51,52,53]. Because isolated pantothenic acid deficiency is uncommon in humans, the clinical relevance of B5 in neuropathy remains primarily mechanistic rather than supported by direct interventional evidence. Altered pantothenate or CoA-related metabolism has been described in selected neurodegenerative contexts, but these findings should not be generalized to routine neuropathy management without further clinical evidence [54,55].

### 5.5. B6/Pyridoxine and PLP

B6/pyridoxine is converted intracellularly to its active coenzyme form, pyridoxal 5′-phosphate (PLP), which serves as an essential cofactor for over 140 enzymatic reactions involving the metabolism of glucose, lipids, and amino acids, as well as in neurotransmitter synthesis. Although B6 does not directly catalyze TCA cycle reactions, PLP plays a critical role in feeding amino acid-derived carbon skeletons into the cycle through transamination reactions—most importantly the conversion of aspartate to oxaloacetate, a key anaplerotic reaction that replenishes TCA cycle intermediates. In the nervous system, PLP is the cofactor for aromatic L-amino acid decarboxylase (catalyzing synthesis of serotonin and dopamine) and glutamate decarboxylase (catalyzing GABA synthesis).

A notable clinical feature unique to B6 among the B vitamins is that both deficiency and excess can cause peripheral neuropathy [56,57,58]. In deficiency, a length-dependent peripheral neuropathy begins at the feet and ascends the lower limbs, with burning pain, numbness, paresthesias, and reduction in deep tendon reflexes [42]. Conversely, high B6 intake from supplements may lead to a predominantly sensory neuropathy. The most typical pattern is a sensory ganglionopathy (or neuronopathy), which is non-length-dependent, mediated by direct toxicity to dorsal root ganglia cells [57,58]. This dual risk underscores that B6 should be discussed as a nutrient requiring careful dose consideration rather than as a uniformly benign component of supplementation. Evidence for B6 as monotherapy in neuropathy remains limited, and available clinical discussions usually involve deficiency correction or combination regimens rather than isolated B6 treatment.

### 5.6. B9/Folate

B9/folate is required for the synthesis of pyrimidines and purines essential for DNA replication and cell mitosis, and plays a crucial role in nervous system repair in adults. Folate-derived tetrahydrofolate (THF) cycles through the folate cycle to provide one-carbon units required for nucleotide synthesis and, critically, for the remethylation of homocysteine to methionine in the methionine cycle—a reaction that also depends on B12/cobalamin and B2/riboflavin. B9/folate deficiency may contribute to homocysteine accumulation and reduced SAM availability, thereby affecting methylation-dependent processes relevant to myelin maintenance.

A key interaction between folate and other B vitamins is the “folate trap” phenomenon: B12 deficiency impairs methionine synthase activity, leading to trapping of folate as methyltetrahydrofolate and resulting in functional folate deficiency with increased urinary folate loss even when dietary folate intake is adequate [11,45]. B9/folate deficiency neuropathy may present as a predominantly sensory neuropathy with slow progression, affecting deep sensation more than superficial sensation, and has been reported as a risk factor for neuropathy in selected populations [59,60,61]. Clinical interpretation of folate status should be integrated with B12 status, because folate supplementation alone may mask hematological manifestations of B12 deficiency while neurological injury progresses.

### 5.7. B12/Cobalamin

B12/cobalamin participates in two major enzymatic reactions in the nervous system that directly intersect with the TCA cycle. In the mitochondria, adenosylcobalamin (AdoCbl) serves as a cofactor for methylmalonyl-CoA mutase (MCM), which catalyzes the conversion of L-methylmalonyl-CoA to succinyl-CoA—a key TCA cycle intermediate—derived from the catabolism of odd-chain fatty acids, certain amino acids, and cholesterol. In the cytosol, methylcobalamin (MeCbl) is required by methionine synthase (MS) to convert homocysteine to methionine, which is essential not only for protein and neurotransmitter synthesis but also as a precursor to SAM, crucial for maintaining myelin sheaths.

B12/cobalamin deficiency is among the better-established nutritional causes of combined peripheral and central neurological manifestations, including myelopathy, peripheral neuropathy, optic neuropathy, cognitive changes, and, less commonly, psychiatric presentations [10,11]. B12/cobalamin deficiency-related neuropathy is related to impaired myelin maintenance and accumulation of metabolites such as homocysteine and methylmalonic acid, which trigger inflammation, oxidative stress, and microvascular disease [12,62,63]. The most common finding in B12/cobalamin deficiency-related neuropathy is chronic axonal sensory polyneuropathy, and neurological sequelae may take 2–5 years to manifest due to hepatic reserves [10,50,60].

## 6. Biochemical Interdependence of B Vitamins and the Concept of Combined Deficiency

The concept of combined deficiency is clinically relevant in nutritional neuropathy because poor intake, malabsorption, alcohol exposure, bariatric surgery, and chronic medication exposure may affect multiple B vitamins simultaneously [4,17]. Biochemical interdependence supports assessment for coexisting deficiencies in selected clinical contexts, but it does not imply that all patients with neuropathy require broad B vitamin supplementation. Specific situations—such as alcoholism—are favorable to the development of multiple B vitamin deficiencies simultaneously, with B1/thiamine deficiency reported in 30–80% of alcoholics, B6-related deficiency in 50%, and B12/cobalamin deficiency in 17% [64,65]. Metabolically, several B vitamins rely on the biologically active forms of B2/riboflavin in order to function properly; a B2 deficiency could therefore give rise to secondary deficiencies in folate, B6/PLP-dependent function and B12/cobalamin metabolism [66,67].

The interdependence of B vitamins is vividly illustrated by their collective contributions to the TCA cycle and linked folate and methionine cycles. The pyruvate dehydrogenase complex alone requires B1/thiamine as TPP, B2/riboflavin as FAD, B3/niacin as NAD^+^, and B5/pantothenic acid as CoA, and B6 for transamination reactions feeding substrates into the cycle [18]. Downstream, riboflavin (FAD) is required for succinate dehydrogenase, niacin (NAD^+^) for isocitrate dehydrogenase, α-ketoglutarate dehydrogenase, and malate dehydrogenase, pantothenate (CoA) for succinyl-CoA synthetase, and cobalamin (AdoCbl) for the anaplerotic conversion of methylmalonyl-CoA to succinyl-CoA [46,51].

B1/thiamine, B6/PLP, and B12/cobalamin have non-substitutable biochemical roles in neural metabolism, including energy production, amino acid and neurotransmitter metabolism, and myelin maintenance [20,22,68]. Preclinical data suggest potential complementary effects of combined B1, B6, and B12 administration in experimental diabetic neuropathy models [69]. However, these findings should be interpreted as hypothesis-generating and should not be directly extrapolated to routine broad supplementation in all patients with neuropathy.

## 7. Anaplerotic Pathways and Interdependence in the TCA Cycle

In addition to the core TCA cycle reactions, anaplerotic pathways—reactions that replenish depleted cycle intermediates—also involve several vitamin-dependent enzymes. The most prevalent anaplerotic pathway involves the augmentation of succinyl-CoA from α-ketobutyrate generated from methionine within the methionine cycle, a process that requires B12/cobalamin as adenosylcobalamin via methylmalonyl-CoA mutase, biotin, and branched-chain amino acid catabolism involving B1/thiamine, B2/riboflavin, B3/niacin, and B6-related coenzymes [18,70,71]. A second major anaplerotic pathway involves the direct synthesis of oxaloacetate from pyruvate by pyruvate carboxylase, which requires biotin (B7), underscoring that even vitamins less often discussed in the neuropathy literature contribute to the maintenance of TCA cycle flux [70,72].

Impairment of anaplerotic pathways due to deficiency of any of these vitamins reduces the availability of TCA intermediates, not only diminishing energy production but also limiting the synthesis of amino acids, fatty acids (including those required for myelin), and nucleotides that are generated from cycle intermediates [23,62]. In neurons, whose metabolic demands are exceptionally high and whose regenerative capacity is limited, impairment of these pathways may contribute to axonal vulnerability, altered myelin maintenance, and neuropathic manifestations, particularly when combined with other nutritional or metabolic stressors [4,19,73].

## 8. Metabolic Cycle Interconnections: TCA Cycle, Urea Cycle, and the MTHFR-Linked Folate/Methionine Cycle

### 8.1. The Urea Cycle–TCA Cycle Interface

The TCA cycle does not operate in isolation; it is metabolically coupled to the urea cycle via shared intermediates and B6-dependent transaminase reactions—an interconnection functionally linking the two pathways via the aspartate–argininosuccinate shunt. The key bridging metabolites are fumarate and aspartate. Fumarate, a TCA cycle intermediate produced by fumarase, is also a direct byproduct of the urea cycle’s argininosuccinate lyase reaction—the step converting argininosuccinate to arginine and fumarate. This fumarate re-enters the TCA cycle and is hydrated to malate, then oxidized to oxaloacetate, replenishing TCA cycle intermediates in a major anaplerotic contribution from the urea cycle [18]. Conversely, the urea cycle depends on aspartate to incorporate the second nitrogen destined for urea, and aspartate is synthesized from oxaloacetate (a TCA cycle intermediate) via the aspartate aminotransferase reaction, which is entirely dependent on B6-derived PLP as a cofactor [74]. Similarly, glutamate—the primary nitrogen donor to the urea cycle—is regenerated from α-ketoglutarate (also a TCA cycle intermediate) by B6-dependent aminotransferases [74]. These interactions illustrate a biochemical connection between nitrogen handling and mitochondrial metabolism, with B6/PLP serving as an important coenzyme at this interface [75].

### 8.2. The MTHFR-Linked Folate/Methionine Cycle and Its Intersection with the TCA Cycle

The folate cycle and methionine cycle are closely interlinked with the TCA cycle through two principal enzymatic intersections. First, MTHFR catalyzes the irreversible reduction of 5,10-methylenetetrahydrofolate to 5-methyltetrahydrofolate (5-MTHF), the form of folate required for homocysteine remethylation to methionine. MTHFR is a flavoenzyme that requires FAD (B2/riboflavin) as an obligate cofactor; its activity is therefore directly dependent on B2 status, linking folate cycle flux to mitochondrial flavin homeostasis [26]. Second, the methionine synthase reaction requires B12-derived methylcobalamin as a cofactor, and the resulting methionine serves as a precursor to SAM, the universal methyl donor for myelin synthesis, DNA methylation, and neurotransmitter metabolism [6,45]. Methionine catabolism also connects back to the TCA cycle: methionine → SAM → homocysteine → cystathionine → α-ketobutyrate → propionyl-CoA → succinyl-CoA (TCA intermediate), a pathway dependent on B6/PLP-related transsulfuration enzymes and B12/cobalamin-dependent methylmalonyl-CoA mutase. This network illustrates how B2/riboflavin, B6/PLP, B9/folate, and B12/cobalamin converge in one-carbon metabolism and TCA-related anaplerotic flux.

### 8.3. MTHFR Polymorphisms as a Mechanistic Example of Riboflavin-Dependent One-Carbon Metabolism

The gene encoding MTHFR is subject to two well-characterized common single nucleotide polymorphisms (SNPs) that reduce enzymatic activity: C677T (rs1801133) and A1298C (rs1801131) [76,77]. The C677T variant encodes an Ala222Val substitution that reduces FAD cofactor binding affinity, causing the enzyme to be thermolabile; homozygous TT individuals retain only approximately 20% of wild-type MTHFR residual activity, while heterozygous CT individuals retain approximately 56% [26]. This reduced FAD affinity means that MTHFR activity in TT individuals is highly sensitive to B2/riboflavin status: under low riboflavin intake, TT individuals lose substantially more enzyme activity, resulting in elevated plasma homocysteine concentrations [26].

These polymorphisms are extremely common globally. According to the 1000 Genomes Project, approximately 25% of the global population carry the MTHFR 677C>T allele, with the highest frequency in Hispanics (47%), followed by Europeans (36%), East Asians (30%), South Asians (12%), and Africans (9%) [78,79,80]. Approximately 13.5% of Europeans are homozygous TT. The MTHFR 1298A>C polymorphism affects approximately 25% of the global population, with the highest frequency in South East Asians (42%) and Europeans (31%) [79,81]. In primary care cohorts, patients carrying at least one variant allele at either locus frequently exceed 50–60% of the population [44,81]. Notably, diabetic peripheral neuropathy and retinopathy occur more frequently among individuals with hyperhomocysteinemia driven by MTHFR SNPs, and a meta-analysis confirmed a significant association between MTHFR 677C>T and diabetic peripheral neuropathy [82,83].

These findings have mechanistic relevance but should not be interpreted as indicating that MTHFR genotype alone should guide supplementation. Common MTHFR variants are highly prevalent, and their clinical significance depends on biochemical context, including folate status, B12 status, homocysteine, methylmalonic acid, diet, comorbidities, medication exposure, and neurological phenotype. Therefore, MTHFR polymorphisms are best interpreted as one example of how B2/riboflavin-dependent enzyme function may influence one-carbon metabolism, rather than as a stand-alone indication for genetic testing or genotype-guided supplementation [43,44,45]. Consistent with this cautious interpretation, professional guidance has questioned the clinical utility of routine MTHFR polymorphism testing in several settings; therefore, MTHFR genotyping should not be used as a substitute for biochemical assessment or clinical evaluation [84].

## 9. Dietary Reference Intakes, Safety Limits, and Context-Specific Repletion Considerations

Understanding recommended intakes, safety limits, and clinically used repletion ranges is important for interpreting B vitamin biology in a clinically responsible manner. However, dietary reference values, tolerable upper intake levels, pharmacological doses, and physician-supervised repletion regimens should be distinguished. Table 1 summarizes adult dietary reference intakes and safety limits, whereas Table 2 summarizes selected clinical repletion contexts and evidence considerations. These ranges should not be interpreted as universal supplementation recommendations.

Several safety considerations warrant emphasis. For B6/pyridoxine, the EFSA lowered the adult tolerable upper intake level to 12 mg/day based on concerns regarding peripheral neuropathy risk, whereas the US UL remains higher. This discrepancy highlights the need to distinguish population-level safety limits from physician-supervised treatment in selected deficiency or drug-induced risk states. For B12/cobalamin, no UL has been established, but delayed treatment of clinically significant deficiency may lead to incomplete neurological recovery. Therefore, dosing decisions should be individualized according to deficiency severity, absorption status, neurological phenotype, and functional biomarkers [85].

## 10. Adjunctive Nutrients with Mitochondrial or Antioxidant Relevance: L-Carnitine and Alpha-Lipoic Acid

### 10.1. L-Carnitine: Bridging Fatty Acid Oxidation and the TCA Cycle

L-carnitine (β-hydroxy-γ-trimethylaminobutyric acid) is a quaternary amine synthesized in the liver and kidney from lysine and methionine, or obtained from dietary sources (predominantly red meat and dairy) [86]. Its principal biochemical role is to transport long-chain fatty acids across the inner mitochondrial membrane via the carnitine palmitoyltransferase (CPT) system, enabling their entry into the mitochondrial matrix for β-oxidation. β-oxidation of fatty acids sequentially cleaves two-carbon units as acetyl-CoA, which then enters the TCA cycle directly via condensation with oxaloacetate in the citrate synthase reaction. In metabolically active neural tissues, fatty acid oxidation may contribute to mitochondrial substrate availability alongside glucose-derived metabolism. In this context, L-carnitine availability may influence fatty-acid-derived acetyl-CoA supply to the TCA cycle [86,87]. L-carnitine also exists in an acetylated form, acetyl-L-carnitine (ALC), which can directly donate acetyl groups for acetylcholine synthesis—the principal neurotransmitter at the neuromuscular junction—making it additionally relevant to peripheral nerve function. The carnitine shuttle system (CPT-I/CACT/CPT-II) enables the transport of long-chain fatty acids into the mitochondrial matrix, thereby linking fatty acid β-oxidation to acetyl-CoA production and subsequent entry into the TCA cycle.

Although free carnitine is structurally regenerated at the CPT-II step, the total body carnitine pool is subject to continuous depletion through renal excretion of both free carnitine and acylcarnitine species [88,89,90]. Moreover, endogenous L-carnitine biosynthesis—which requires lysine, methionine, and cofactors including B3/niacin, B6-related coenzymes, and ascorbate—may be secondarily impaired when B vitamin status is inadequate, as methionine availability itself depends on the B12/cobalamin- and B9/folate-dependent methionine cycle [91,92]. These mechanisms provide a biochemical rationale for considering carnitine status in selected conditions of nutritional deficiency, increased metabolic demand, chronic kidney disease, or impaired biosynthetic capacity, but they do not establish a general requirement for L-carnitine supplementation in all patients with neuropathy [93,94,95].

Potential metabolic complementarity between L-carnitine and B vitamin-dependent pathways arises at several biochemical junctions. The acetyl-CoA generated by L-carnitine-facilitated β-oxidation enters the TCA cycle, where its efficient oxidation depends on B vitamin-derived coenzymes, including NAD^+^ from B3/niacin, FAD from B2/riboflavin, CoA from B5/pantothenic acid, and TPP from B1/thiamine [23,75,87]. Conversely, without adequate B vitamin coenzyme support, the accumulation of TCA intermediates can impair the regeneration of free CoA, secondarily reducing carnitine-dependent fatty acid transport. Furthermore, the methionine required for L-carnitine biosynthesis is linked to the B12/cobalamin- and B9/folate-dependent methionine cycle, suggesting a biochemical connection between L-carnitine availability and B vitamin-dependent methionine metabolism [45,88]. In a double-blind, placebo-controlled randomized trial of patients with type 2 diabetes and established peripheral neuropathy, a multi-component regimen including ALC, alpha-lipoic acid, SOD, and B12/cobalamin improved several neuropathy-related indices compared with placebo. However, because the intervention combined multiple components, the independent contribution of ALC cannot be isolated. A meta-analysis of randomized trials reported improvements in selected neuropathic outcomes with ALC, including pain and vibration perception threshold; however, the included studies varied in dose, duration, and outcome measures, limiting generalizability beyond the studied diabetic neuropathy populations [96].

### 10.2. Alpha-Lipoic Acid: Structural Partner of Dehydrogenase Complexes and Antioxidant Adjunct

Alpha-lipoic acid (ALA; 1,2-dithiolane-3-pentanoic acid) occupies a unique position among nutrients: it is not merely a cofactor but a structural component of the multienzyme pyruvate dehydrogenase (PDH) complex, covalently attached (as lipoamide) to the E2 dihydrolipoyl transacetylase subunit. Within the PDH complex, the lipoamide moiety of ALA acts as a swinging arm, transferring the acetyl group from B1/thiamine-derived TPP on the E1 subunit to B5/pantothenic acid-derived CoA on the E3 subunit, while being simultaneously reduced and re-oxidized by B2/riboflavin-derived FAD on the E3 (dihydrolipoyl dehydrogenase) subunit. ALA is therefore a structural and functional partner of B1/thiamine, B2/riboflavin, and B5/pantothenic acid at a major entry point into the TCA cycle, and its depletion—or its replacement by oxidatively damaged lipoamide—would impair the PDH complex independently of B vitamin availability [97,98]. The same lipoamide-containing multienzyme architecture is present in the α-ketoglutarate dehydrogenase complex (α-KGDH) within the TCA cycle, indicating that lipoate participates in two major mitochondrial dehydrogenase control points.

Unlike the freely recycled B vitamin coenzymes (NAD^+^, FAD, CoA, TPP), ALA is not released from the enzyme complex after each catalytic cycle but remains covalently tethered to the E2 subunit as lipoamide. Its replenishment is therefore driven not by urinary loss but by the continuous biosynthetic demand accompanying protein turnover of the PDH and α-KGDH complexes, and by irreversible oxidative inactivation of the lipoamide moiety under the conditions of oxidative stress that characterize nutritional and diabetic neuropathy [97,98,99]. Under conditions of excessive reactive oxygen species, lipoamide may undergo irreversible oxidative modification. In its free circulating form, ALA also functions as an antioxidant capable of scavenging reactive oxygen species and regenerating vitamins C and E and glutathione from their oxidized forms—a function that protects nerve tissue from the oxidative stress that characterizes nutritional neuropathy and diabetic neuropathy alike [97,100]. ALA improves insulin sensitivity and glucose uptake in peripheral tissues, potentially reducing the hyperglycemia-mediated oxidative damage to nerves [101,102]. Crucially, as an antioxidant, ALA can maintain the reduced state of lipoamide within the PDH and α-KGDH complexes, preventing their oxidative inactivation and thereby preserving TCA cycle activity in oxidative stress conditions—a function that may complement B vitamin-dependent enzyme activity under oxidative stress conditions [18,97]. Clinical trials and meta-analyses suggest that alpha-lipoic acid may improve selected neuropathic symptoms and electrophysiological outcomes in diabetic polyneuropathy, particularly at commonly studied doses around 600 mg/day. However, findings are not uniform across trials, and the evidence is more directly applicable to diabetic neuropathy than to nutritional neuropathy or neuropsychiatric outcomes [103,104,105,106].

ALA, L-carnitine, and B vitamin-dependent pathways may be considered metabolically complementary in selected contexts: ALA and B vitamins share structural and functional roles within the PDH and α-KGDH complexes; L-carnitine supports acetyl-CoA delivery through fatty acid oxidation; ALC provides acetyl groups relevant to acetylcholine synthesis; and ALA may protect B vitamin-dependent enzyme complexes and neural membrane lipids from oxidative stress. Clinical evidence for these components is strongest for ALA and ALC in diabetic neuropathy and selected entrapment neuropathy contexts, whereas direct evidence for fixed multi-nutrient combinations remains more limited and context-dependent [96,107,108].

## 11. Clinical Implications: Assessment of B Vitamin Status in Neural Vulnerability

### 11.1. Clinical Evaluation of Combined B Vitamin Deficiency

In clinical practice, the identification of a single low B vitamin level should prompt evaluation for coexisting deficiencies, because the metabolic interdependencies described above suggest that poly-deficiency is common in nutritional neuropathy [4,5,17]. Serum B12/cobalamin and B9/folate levels should be interpreted together because of their overlapping roles in one-carbon metabolism and the possibility of combined deficiency [1,10,11]. Functional metabolites such as homocysteine and methylmalonic acid can help identify metabolic deficiency even when direct vitamin levels are borderline, particularly in patients with neurological symptoms, older age, diabetes, restrictive diets, bariatric surgery, gastrointestinal disease, or chronic medication exposure [12,109,110].

This approach is important because serum vitamin concentrations do not always reflect intracellular coenzyme availability [10,12,110]. For example, mitochondrial redox imbalance may functionally limit the NAD^+^/NADH or FAD/FADH2 cycling required for TCA cycle flux, while impaired cobalamin-dependent methylmalonyl-CoA mutase activity may elevate methylmalonic acid despite variable serum B12 levels. Clinical assessment should therefore integrate dietary history, gastrointestinal risk factors, medication exposure, neurological phenotype, and functional biomarkers rather than relying on a single circulating vitamin value.

### 11.2. Neuropsychiatric Relevance: Variable Strength of Evidence Across Outcomes

Although this review focuses primarily on nutritional neuropathy, B vitamin-dependent coenzyme pathways are also relevant to central neural function [6,8,9]. The strength of evidence linking B vitamin status to neuropsychiatric outcomes differs substantially by condition. Cognitive impairment, delirium, myelopathy, and neuropathy are more clearly described in established B12 or thiamine deficiency states. Associations between folate, B12, homocysteine, and depressive symptoms are biologically plausible and supported by observational evidence, but interventional findings remain heterogeneous and may depend on baseline deficiency, age, comorbidities, and homocysteine status. Psychosis-like presentations have been reported in severe deficiency states, particularly B12 deficiency, but these are uncommon and should not be generalized to primary psychiatric disorders. Evidence for fatigue and sleep-related symptoms is more indirect and may reflect multiple nutritional, endocrine, inflammatory, psychiatric, or medication-related factors. Therefore, neuropsychiatric symptoms should be considered a reason to assess nutritional and metabolic risk in appropriate clinical contexts, not as a stand-alone indication for broad B vitamin supplementation.

B6/PLP provides an additional connection because pyridoxal 5′-phosphate is required for neurotransmitter-related reactions involving serotonin, dopamine, γ-aminobutyric acid, and glutamate metabolism. Both deficiency and excess of B6 may have neurological consequences: deficiency may impair neurotransmitter synthesis and amino acid metabolism, whereas chronic high-dose pyridoxine exposure can produce sensory neuronopathy or peripheral neuropathy [56,57,58,111]. This duality supports careful, context-specific repletion rather than indiscriminate high-dose use.

### 11.3. Medication-Associated Limitation of B Vitamin Availability

B vitamin deficiency should not be interpreted solely as a dietary problem. Chronic medication exposure may reduce B vitamin availability through impaired gastric release or intestinal absorption, interference with receptor-mediated uptake, increased urinary loss, vitamin antagonism, hepatic enzyme induction, accelerated catabolism, or increased methylation demand [3]. These mechanisms are clinically relevant in patients with neuropathy, diabetes, psychiatric disease, cardiovascular disease, chronic pain, or polypharmacy [3]. Metformin is a clinically important example because long-term use is associated with B12/cobalamin deficiency and may worsen or mimic neuropathic symptoms in patients with diabetes [3]. Gastric acid suppressive therapy, including proton pump inhibitors and histamine-2 receptor antagonists, may impair the release of food-bound cobalamin from dietary protein [3,112].

Isoniazid is a well-established cause of B6/pyridoxine-responsive neuropathy, while methotrexate and other antifolate agents interfere with folate-dependent pathways [3,113,114]. Enzyme-inducing antiepileptic drugs may reduce folate or pyridoxal 5′-phosphate status, levodopa metabolism may increase homocysteine and alter B6/PLP, B12/cobalamin, and B9/folate metabolism, and loop diuretics such as furosemide may increase urinary thiamine loss [3,115,116,117]. Representative medication-associated mechanisms are summarized in Table 3.

### 11.4. Context-Specific Repletion in Patients with Deficiency or High-Risk Features

When B vitamin deficiency or functional insufficiency is documented or strongly suspected, the clinical goal of repletion is to restore adequate biochemical function while avoiding unnecessary high-dose exposure. Evidence from mechanistic, preclinical, and selected clinical sources suggests that B1, B6, and B12 have complementary roles in neural function, but this does not establish that fixed high-dose combinations are necessary or superior in all patients with neuropathy [7,22,69]. In particular, chronic high-dose pyridoxine exposure is associated with sensory neuropathy; therefore, B6-containing regimens should be dose-conscious and clinically supervised [56,57,58,85].

In patients with established neuropathy and relevant nutritional, gastrointestinal, metabolic, medication-related, or functional biomarker abnormalities, repletion should be individualized according to clinical context. Patients with B12/cobalamin deficiency may require high-dose oral or intramuscular B12 depending on severity and absorption status [10,118]. Patients with suspected one-carbon metabolism impairment may require assessment of B9/folate, B12/cobalamin, homocysteine, and methylmalonic acid, with careful avoidance of folate monotherapy in unrecognized B12/cobalamin deficiency. In patients with diabetes or metabolic neuropathy, oxidative stress-related and mitochondrial factors may be relevant to assessment. Adjunctive nutrients such as alpha-lipoic acid and acetyl-L-carnitine have been studied mainly in selected metabolic neuropathy contexts and should be discussed as indication-specific adjuncts rather than routine components of B vitamin repletion [96,100,102,103]. Patients taking medications known to impair B vitamin availability should be assessed not only for absolute deficiency but also for functional biochemical markers and compatible neurological phenotypes [3].

When a clinically relevant deficiency is promptly identified and corrected, nutritional neuropathies may stabilize or improve, although the degree of recovery depends on deficiency severity, duration, and underlying comorbidities. However, if deficiencies go unrecognized—particularly B12/cobalamin, B9/folate, or B1/thiamine deficiencies—neurological and neuropsychiatric deficits may become only partially reversible. Therefore, in patients with neuropathy and relevant nutritional, gastrointestinal, metabolic, or medication-related risk factors, B vitamin status may be interpreted as part of a broader metabolic assessment rather than as an isolated laboratory value.

## 12. Evidence Summary

Table 4 summarizes the types and relative strength of evidence supporting the principal B-vitamin-related neural outcomes and adjunctive interventions discussed in this review. The evidence is strongest for correction of established thiamine and cobalamin deficiency states, whereas support for fixed B-vitamin combinations, genotype-guided supplementation, and adjunctive nutrients remains heterogeneous and context-dependent.

## 13. Limitations

This review has several limitations. First, because it is a narrative review, the literature search and synthesis were not designed to provide the level of evidence certainty expected from a systematic review or meta-analysis. Second, many relationships described in this article are based on established biochemical mechanisms, but mechanistic plausibility does not necessarily translate into clinical efficacy. Third, clinical evidence for broad or fixed multi-nutrient B vitamin combinations remains limited and heterogeneous, with variability in patient populations, baseline deficiency status, formulations, doses, treatment duration, and outcome measures. Fourth, evidence differs substantially across neurological and neuropsychiatric outcomes; findings from deficiency states should not be generalized to patients without documented deficiency or relevant risk factors. Fifth, the clinical relevance of common MTHFR polymorphisms remains context-dependent, and genotype alone should not be used as a stand-alone indication for supplementation. Finally, adjunctive nutrients such as alpha-lipoic acid and acetyl-L-carnitine have more evidence in selected metabolic neuropathies than in nutritional neuropathy or neuropsychiatric disorders, and their role in fixed combination regimens requires further study.

## 14. Conclusions

The B vitamin complex forms an interconnected coenzyme network that supports mitochondrial energy metabolism, one-carbon metabolism, neurotransmitter synthesis, redox regulation, and myelin maintenance. This mechanistic framework may help explain why nutritional neuropathy and selected neuropsychiatric manifestations occur in patients with dietary restriction, malabsorption, aging-related vulnerability, metabolic disease, or chronic medication exposure. However, biochemical interdependence does not by itself establish the clinical efficacy of broad B vitamin supplementation. Clinical evaluation and repletion should be guided by neurological phenotype, dietary and gastrointestinal risk factors, medication history, documented deficiency, and functional biomarkers such as homocysteine and methylmalonic acid. Future well-designed clinical studies are needed to determine whether correction of combined or functional B vitamin insufficiency improves neurological or neuropsychiatric outcomes beyond established deficiency states.

## Figures and Tables

**Table 1:** Adult dietary reference intakes and tolerable upper intake levels for B vitamins.

Vitamin	Active Coenzyme Form	Adult RDA/AI	US UL	EFSA UL	Safety Consideration
B1	Thiamine pyrophosphate (TPP)	1.1–1.2 mg/day	Not established	Not established	No established UL; toxicity is uncommon at standard oral doses
B2	FAD, FMN	1.1–1.3 mg/day	Not established	Not established	No established UL; excess may cause bright yellow urine
B3	NAD^+^, NADP^+^	14–16 mg NE/day	35 mg/day for nicotinic acid	Not established for nicotinamide	Flushing and hepatotoxicity risk are mainly relevant to pharmacological nicotinic acid doses
B5	Coenzyme A	5 mg/day AI	Not established	Not established	No established UL; very high doses may cause gastrointestinal discomfort
B6	Pyridoxal 5′-phosphate (PLP)	1.3–1.7 mg/day	100 mg/day	12 mg/day	Chronic high-dose exposure may cause sensory neuropathy
B9	THF derivatives, 5-MTHF	400 μg DFE/day	1000 μg/day synthetic folic acid	1000 μg/day	High folic acid intake may mask B12 deficiency; interpret with B12 status
B12	Methylcobalamin, adenosylcobalamin	2.4 μg/day	Not established	Not established	No established UL; neurological injury may be partially irreversible if deficiency treatment is delayed

Abbreviations: AI, adequate intake; DFE, dietary folate equivalents; EFSA, European Food Safety Authority; FAD, flavin adenine dinucleotide; FMN, flavin mononucleotide; NE, niacin equivalents; RDA, recommended dietary allowance; THF, tetrahydrofolate; UL, tolerable upper intake level; US, United States.

**Table 2:** Clinical repletion ranges and evidence considerations for B vitamins in neurological contexts.

Vitamin	Clinical Context	Repletion Range	Evidence Context	Caution
B1	Deficiency, alcohol exposure, Wernicke-risk states, nutritional neuropathy	Oral 25–100 mg/day; parenteral dosing for acute severe deficiency	Strong for deficiency syndromes	Do not delay parenteral therapy when Wernicke encephalopathy is suspected
B2	Riboflavin deficiency; FAD-dependent one-carbon metabolism context	10–100 mg/day in selected clinical contexts; higher doses have been used for non-neuropathy indications	Mechanistic and selected clinical contexts	MTHFR genotype alone should not dictate supplementation
B3	Deficiency prevention or pellagra	25–100 mg/day nicotinamide in selected contexts	Strong for pellagra; limited for neuropathy-specific use	Pharmacological lipid-lowering niacin doses are a separate indication
B5	Rare deficiency or combined nutritional deficiency	10–100 mg/day in selected contexts	Mostly mechanistic; limited direct neuropathy evidence	Isolated deficiency is uncommon
B6	Isoniazid exposure, deficiency, selected drug-induced risk states	Dose should be individualized; physician-supervised short-term use may be appropriate in selected drug-induced deficiency contexts	Strong for isoniazid-related prevention; caution for broad use	Chronic high-dose pyridoxine can cause neuropathy
B9	Folate deficiency, hyperhomocysteinemia context	0.4–1 mg/day commonly used for prevention or repletion; higher doses under physician supervision	Strong for deficiency; mixed for neuropsychiatric outcomes	Avoid folate monotherapy if B12 deficiency is possible; MTHFR genotype alone should not determine folate formulation
B12	Confirmed or suspected deficiency, malabsorption, metformin/PPI exposure, neuropathy/myelopathy	Oral 500–1000 μg/day or intramuscular regimens depending on severity and absorption status	Strong for deficiency-related neuropathy/myelopathy	Serum B12 may not fully reflect functional deficiency; consider MMA and homocysteine

The ranges shown in Table 2 summarize commonly used clinical repletion ranges reported in selected deficiency or high-risk contexts. They should not be interpreted as universal supplementation recommendations. Individual treatment should be guided by documented deficiency, clinical severity, absorption status, medication exposure, functional biomarkers, comorbidities, and physician supervision.

**Table 3:** Medication-associated limitations of B vitamin availability and their relevance to neural dysfunction.

Mechanism	Medication	Vitamin	Evidence Strength	Clinical Consideration
Impaired absorption	Metformin	B12/cobalamin	Moderate–strong	Long-term use, anemia, neuropathy
Reduced gastric release	PPI/H2 blocker	B12/cobalamin	Moderate	Older adults, malnutrition, neurologic symptoms
Vitamin antagonism	Isoniazid	B6/pyridoxine	Strong	Prophylactic pyridoxine in high-risk patients
Antifolate effect	Methotrexate	B9/folate	Strong by indication	Folate/folinic acid per protocol
Enzyme induction	Phenytoin/carbamazepine	B9/folate and B6/PLP	Moderate	Long-term therapy, homocysteine elevation
Increased urinary loss	Furosemide	B1/thiamine	Limited–moderate	High-risk heart failure, poor intake
Increased methylation demand	Levodopa	B6/PLP, B9/folate, and B12/cobalamin	Moderate	Neuropathy or high-dose chronic exposure

**Table 4:** Summary of evidence strength for B vitamin-related neural outcomes and interventions.

Topic	Evidence Type	Strength	Interpretation
B12 deficiency neuropathy/myelopathy	Clinical, biochemical, guideline-based	Strong	Treat confirmed or strongly suspected deficiency
Thiamine deficiency neurological syndromes	Clinical deficiency syndrome	Strong	Prompt repletion in high-risk deficiency states
B6 deficiency neuropathy	Clinical/pharmacological	Moderate	Replete deficiency; avoid chronic excess
B1/B6/B12 combination for neuropathic symptoms	Preclinical data and limited clinical consensus	Low–moderate	Consider only in selected deficient or high-risk contexts; not a universal neuropathy strategy
MTHFR genotype-guided supplementation	Mechanistic, heterogeneous clinical relevance	Low/controversial	Genotype alone should not guide therapy
B vitamins and neuropsychiatric outcomes	Observational studies, deficiency-state reports, and heterogeneous interventional data	Variable	Interpret separately by outcome; assess deficiency risk and avoid causal overstatement
ALA in diabetic neuropathy	RCTs/meta-analyses with variable results	Moderate	Indication-specific adjunct; evidence should not be generalized to nutritional neuropathy
ALC in diabetic neuropathy	RCTs/meta-analyses with heterogeneous regimens	Moderate/limited	Potential adjunct in selected populations; heterogeneous data limit generalizability

## Data Availability

No new data were created or analyzed in this study. Data sharing is not applicable to this article.

## References

[1] Alvarez M., Poveda S., Cisneros A., Parra D., Luna M., Rincón O., Guzman I. (2026). B Vitamin Deficiencies and Associated Neuropathies. Curr. Nutr. Rep..

[2] Kramarz C., Murphy E., Reilly M.M., Rossor A.M. (2023). Nutritional peripheral neuropathies. J. Neurol. Neurosurg. Psychiatry.

[3] Jung J.W., Park S.Y., Kim H. (2022). Drug-Induced Vitamin Deficiency. Ann. Clin. Nutr. Metab..

[4] Kumar N. (2007). Nutritional Neuropathies. Neurol. Clin..

[5] Geller M., Oliveira L., Nigri R. (2017). B Vitamins for Neuropathy and Neuropathic Pain. Vitam. Miner..

[6] Kennedy D.O. (2016). B Vitamins and the Brain: Mechanisms, Dose and Efficacy—A Review. Nutrients.

[7] Cuyubamba O., Braga C.P., Swift D., Stickney J.T., Viel C. (2025). The Combination of Neurotropic Vitamins B1, B6, and B12 Enhances Neural Cell Maturation and Connectivity Superior to Single B Vitamins. Cells.

[8] Bottiglieri T. (1996). Folate, vitamin B12, and neuropsychiatric disorders. Nutr. Rev..

[9] Sahu P., Thippeswamy H., Chaturvedi S.K. (2022). Neuropsychiatric manifestations in vitamin B12 deficiency. Vitam. Horm..

[10] Stabler S.P. (2013). Clinical practice. Vitamin B12 deficiency. N. Engl. J. Med..

[11] O’Leary F., Samman S. (2010). Vitamin B12 in health and disease. Nutrients.

[12] Selhub J. (1999). Homocysteine metabolism. Annu. Rev. Nutr..

[13] American Diabetes Association Professional Practice Committee (2026). Standards of Care in Diabetes—2026. Diabetes Care.

[14] Shankar P., Boylan M., Sriram K. (2010). Micronutrient deficiencies after bariatric surgery. Nutrition.

[15] Jivraj A., Hutchinson J.M., Ching E., Marwaha A., Verdu E.F., Armstrong D., Pinto-Sanchez M.I. (2022). Micronutrient deficiencies are frequent in adult patients with and without celiac disease on a gluten-free diet, regardless of duration and adherence to the diet. Nutrition.

[16] Kim S.E. (2017). Micronutrients Should Be Monitored in the Real Practice for Korean Inflammatory Bowel Disease Patients. Gut Liver.

[17] Hammond N., Wang Y., Dimachkie M.M., Barohn R.J. (2013). Nutritional neuropathies. Neurol. Clin..

[18] Nelson D.L., Cox M.M. (2017). Lehninger Principles of Biochemistry.

[19] Magistretti P.J., Allaman I. (2015). A cellular perspective on brain energy metabolism and functional imaging. Neuron.

[20] Butterworth R.F. (2003). Thiamin deficiency and brain disorders. Nutr. Res. Rev..

[21] Sechi G., Serra A. (2007). Wernicke’s encephalopathy: New clinical settings and recent advances in diagnosis and management. Lancet Neurol..

[22] Calderon-Ospina C.A., Nava-Mesa M.O. (2020). B Vitamins in the nervous system: Current knowledge of the biochemical modes of action and synergies of thiamine, pyridoxine, and cobalamin. CNS Neurosci. Ther..

[23] Depeint F., Bruce W.R., Shangari N., Mehta R., O’Brien P.J. (2006). Mitochondrial function and toxicity: Role of the B vitamin family on mitochondrial energy metabolism. Chem. Biol. Interact..

[24] Wallace D.C. (1999). Mitochondrial diseases in man and mouse. Science.

[25] Schon E.A., Przedborski S. (2011). Mitochondria: The next (neurode)generation. Neuron.

[26] Graydon J.S., Claudio K., Baker S., Kocherla M., Ferreira M., Roche-Lima A., Rodríguez-Maldonado J., Duconge J., Ruaño G. (2019). Ethnogeographic prevalence and implications of the 677C>T and 1298A>C MTHFR polymorphisms in US primary care populations. Biomark. Med..

[27] Hanna M., Jaqua E., Nguyen V., Clay J. (2022). B Vitamins: Functions and Uses in Medicine. Perm. J..

[28] Porter K., Hoey L., Hughes C.F., Ward M., McNulty H. (2016). Causes, Consequences and Public Health Implications of Low B-Vitamin Status in Ageing. Nutrients.

[29] Jeong H., Vacanti N.M. (2020). Systemic vitamin intake impacting tissue proteomes. Nutr. Metab..

[30] Yamada K., Yamada S., Tobimatsu T., Toraya T. (1999). Heterologous high level expression, purification, and enzymological properties of recombinant rat cobalamin-dependent methionine synthase. J. Biol. Chem..

[31] Cellini B., Montioli R., Oppici E., Astegno A., Voltattorni C.B. (2014). The chaperone role of the pyridoxal 5′-phosphate and its implications for rare diseases involving B6-dependent enzymes. Clin. Biochem..

[32] Sun C., Schuman E.M. (2022). Logistics of neuronal protein turnover: Numbers and mechanisms. Mol. Cell. Neurosci..

[33] Moiseenok A.G., Kanunnikova N.P. (2023). Brain CoA and Acetyl CoA Metabolism in Mechanisms of Neurodegeneration. Biochemistry.

[34] Spinelli J.B., Haigis M.C. (2018). The multifaceted contributions of mitochondria to cellular metabolism. Nat. Cell Biol..

[35] Xiao W., Wang R.S., Handy D.E., Loscalzo J. (2018). NAD(H) and NADP(H) Redox Couples and Cellular Energy Metabolism. Antioxid. Redox Signal..

[36] Ying W. (2008). NAD^+^/NADH and NADP^+^/NADPH in cellular functions and cell death: Regulation and biological consequences. Antioxid. Redox Signal..

[37] Stadtman E.R., Levine R.L. (2000). Protein oxidation. Ann. N. Y. Acad. Sci..

[38] Vincent A.M., Callaghan B.C., Smith A.L., Feldman E.L. (2011). Diabetic neuropathy: Cellular mechanisms as therapeutic targets. Nat. Rev. Neurol..

[39] Hotamisligil G.S. (2006). Inflammation and metabolic disorders. Nature.

[40] Nishikawa T., Edelstein D., Du X.L., Yamagishi S.-i., Matsumura T., Kaneda Y., Yorek M.A., Beebe D., Oates P.J., Hammes H.-P. (2000). Normalizing mitochondrial superoxide production blocks three pathways of hyperglycaemic damage. Nature.

[41] Verdin E. (2015). NAD^+^ in aging, metabolism, and neurodegeneration. Science.

[42] Koike H., Sobue G. (2006). Alcoholic neuropathy. Curr. Opin. Neurol..

[43] Powers H.J. (2003). Riboflavin (vitamin B-2) and health. Am. J. Clin. Nutr..

[44] McNulty H., Dowey L.R.C., Strain J.J., Dunne A., Ward M., Molloy A.M., McAnena L.B., Hughes J.P., Hannon-Fletcher M., Scott J.M. (2006). Riboflavin lowers homocysteine in individuals homozygous for the MTHFR 677C->T polymorphism. Circulation.

[45] Stover P.J. (2009). One-carbon metabolism-genome interactions in folate-associated pathologies. J. Nutr..

[46] Bogan K.L., Brenner C. (2008). Nicotinic acid, nicotinamide, and nicotinamide riboside: A molecular evaluation of NAD^+^ precursor vitamins in human nutrition. Annu. Rev. Nutr..

[47] Cantó C., Menzies K.J., Auwerx J. (2015). NAD^+^ Metabolism and the Control of Energy Homeostasis: A Balancing Act between Mitochondria and the Nucleus. Cell Metab..

[48] Houtkooper R.H., Cantó C., Wanders R.J., Auwerx J. (2010). The secret life of NAD^+^: An old metabolite controlling new metabolic signaling pathways. Endocr. Rev..

[49] Hegyi J., Schwartz R.A., Hegyi V. (2004). Pellagra: Dermatitis, dementia, and diarrhea. Int. J. Dermatol..

[50] Jameson J.L., Fauci A.S., Kasper D.L., Hauser S.L., Longo D.L., Loscalzo J. (2022). Harrison’s Principles of Internal Medicine.

[51] Leonardi R., Zhang Y.M., Rock C.O., Jackowski S. (2005). Coenzyme A: Back in action. Prog. Lipid Res..

[52] Asuncion R.M.D., Zulfiqar H., Bokhari S.R.A. (2026). Pantothenate Kinase-Associated Neurodegeneration (PKAN). StatPearls.

[53] Álvarez-Córdoba M., Talaverón-Rey M., Povea-Cabello S., Cilleros-Holgado P., Gómez-Fernández D., Piñero-Pérez R., Reche-López D., Munuera-Cabeza M., Suárez-Carrillo A., Romero-González A. (2023). Patient-Derived Cellular Models for Polytarget Precision Medicine in Pantothenate Kinase-Associated Neurodegeneration. Pharmaceuticals.

[54] Pohane M.R., Dafre R., Sontakke N.G. (2023). Diagnosis and Treatment of Pantothenate Kinase-Associated Neurodegeneration (PKAN): A Systematic Review. Cureus.

[55] Pereira A., Fischinger Moura de Souza C., Álvarez-Córdoba M., Reche-López D., Sánchez-Alcázar J.A. (2024). A therapeutic approach to pantothenate kinase associated neurodegeneration: A pilot study. Orphanet J. Rare Dis..

[56] Parry G.J., Bredesen D.E. (1985). Sensory neuropathy with low-dose pyridoxine. Neurology.

[57] Albin R.L., Albers J.W., Greenberg H.S., Townsend J.B., Lynn R.B., Burke J.M., Alessi A.G. (1987). Acute sensory neuropathy-neuronopathy from pyridoxine overdose. Neurology.

[58] Dalton K., Dalton M.J. (1987). Characteristics of pyridoxine overdose neuropathy syndrome. Acta Neurol. Scand..

[59] Reynolds E.H. (1976). Neurological aspects of folate and vitamin B12 metabolism. Clin. Haematol..

[60] Healton E.B., Savage D.G., Brust J.C., Garrett T.J., Lindenbaum J. (1991). Neurologic aspects of cobalamin deficiency. Medicine.

[61] Choi R., Oh Y., Park M.J., Lee S.G., Lee E.H. (2021). Prevalence of Folate Deficiency in Korean Women of Reproductive Age Using a Serum Folate Assay Traceable to the WHO International Standard. Clin. Lab..

[62] Scalabrino G. (2009). The multi-faceted basis of vitamin B12 (cobalamin) neurotrophism in adult central nervous system: Lessons learned from its deficiency. Prog. Neurobiol..

[63] Tawfik A., Elsherbiny N.M., Zaidi Y., Rajpurohit P. (2021). Homocysteine and Age-Related Central Nervous System Diseases: Role of Inflammation. Int. J. Mol. Sci..

[64] Koike H., Nakamura T., Ikeda S., Takahashi M., Kawagashira Y., Iijima M., Katsuno M., Sobue G. (2017). Alcoholic Myelopathy and Nutritional Deficiency. Intern. Med..

[65] Dudek I., Hajduga D., Sieńko C., Maani A., Sitarz E., Sitarz M., Forma A. (2020). Alcohol-Induced Neuropathy in Chronic Alcoholism: Causes, Pathophysiology, Diagnosis, and Treatment Options. Curr. Pathobiol. Rep..

[66] McCormick D.B. (1989). Two interconnected B vitamins: Riboflavin and pyridoxine. Physiol. Rev..

[67] McNulty H., Strain J.J., Hughes C.F., Ward M. (2017). Riboflavin, MTHFR genotype and blood pressure: A personalized approach to prevention and treatment of hypertension. Mol. Asp. Med..

[68] Gibson G.E., Blass J.P. (2007). Thiamine-dependent processes and treatment strategies in neurodegeneration. Antioxid. Redox Signal..

[69] Jolivalt C.G., Mizisin L.M., Nelson A., Cunha J.M., Ramos K.M., Bonke D., Calcutt N.A. (2009). B vitamins alleviate indices of neuropathic pain in diabetic rats. Eur. J. Pharmacol..

[70] Zempleni J., Hassan Y.I., Wijeratne S.S. (2008). Biotin and biotinidase deficiency. Expert. Rev. Endocrinol. Metab..

[71] Hutson S.M., Sweatt A.J., Lanoue K.F. (2005). Branched-chain amino acid metabolism: Implications for establishing safe intakes. J. Nutr..

[72] Owen O.E., Kalhan S.C., Hanson R.W. (2002). The key role of anaplerosis and cataplerosis for citric acid cycle function. J. Biol. Chem..

[73] Coleman M.P., Freeman M.R. (2010). Wallerian degeneration, wld(s), and nmnat. Annu. Rev. Neurosci..

[74] Parra M., Stahl S., Hellmann H. (2018). Vitamin B_6_ and Its Role in Cell Metabolism and Physiology. Cells.

[75] Murray R.K., Bender D.A., Botham K.M., Kennelly P.J., Rodwell V.W., Weil P.A. (2018). Harper’s Illustrated Biochemistry.

[76] Frosst P., Blom H.J., Milos R., Goyette P., Sheppard C.A., Matthews R.G., Boers G.J., den Heijer M., Kluijtmans L.A., van den Heuvel L.P. (1995). A candidate genetic risk factor for vascular disease: A common mutation in methylenetetrahydrofolate reductase. Nat. Genet..

[77] Lievers K.J., Boers G.H., Verhoef P., den Heijer M., Kluijtmans L.A., van der Put N.M., Trijbels F.J., Blom H.J. (2001). A second common variant in the methylenetetrahydrofolate reductase (MTHFR) gene and its relationship to MTHFR enzyme activity, homocysteine, and cardiovascular disease risk. J. Mol. Med..

[78] Auton A., Brooks L.D., Durbin R.M., Garrison E.P., Kang H.M., Korbel J.O., Marchini J.L., McCarthy S., McVean G.A., Abecasis G.R. (2015). A global reference for human genetic variation. Nature.

[79] Botto L.D., Yang Q. (2000). 5,10-Methylenetetrahydrofolate reductase gene variants and congenital anomalies: A HuGE review. Am. J. Epidemiol..

[80] Zuo M., Lee M.J., Kim M.H., Wu Y., Ayaki H., Nishio H., Sumino K. (1999). C677T mutation of the methylenetetrahydrofolate reductase gene among the Korean infants in Seoul city. Kobe J. Med. Sci..

[81] Wilcken B., Bamforth F., Li Z., Zhu H., Ritvanen A., Renlund M., Stoll C., Alembik Y., Dott B., Czeizel A.E. (2003). Geographical and ethnic variation of the 677C>T allele of 5,10 methylenetetrahydrofolate reductase (MTHFR): Findings from over 7000 newborns from 16 areas world wide. J. Med. Genet..

[82] Hoogeveen E.K., Kostense P.J., Eysink P.E., Polak B.C., Beks P.J., Jakobs C., Dekker J.M., Nijpels G., Heine R.J., Bouter L.M. (2000). Hyperhomocysteinemia is associated with the presence of retinopathy in type 2 diabetes mellitus: The Hoorn study. Arch. Intern. Med..

[83] Diaz-Arrastia R. (2000). Homocysteine and neurologic disease. Arch. Neurol..

[84] Hickey S.E., Curry C.J., Toriello H.V. (2013). ACMG Practice Guideline: Lack of evidence for MTHFR polymorphism testing. Genet. Med..

[85] National Institutes of Health Office of Dietary Supplements Vitamin B6—Health Professional Fact Sheet. https://ods.od.nih.gov/factsheets/VitaminB6-HealthProfessional/.

[86] Bremer J. (1983). Carnitine–metabolism and functions. Physiol. Rev..

[87] McGarry J.D., Brown N.F. (1997). The mitochondrial carnitine palmitoyltransferase system—From concept to molecular analysis. Eur. J. Biochem..

[88] Rebouche C.J., Paulson D.J. (1986). Carnitine metabolism and function in humans. Annu. Rev. Nutr..

[89] Foster D.W. (2004). The role of the carnitine system in human metabolism. Ann. N. Y. Acad. Sci..

[90] Virmani M.A., Cirulli M. (2022). The Role of l-Carnitine in Mitochondria, Prevention of Metabolic Inflexibility and Disease Initiation. Int. J. Mol. Sci..

[91] Vaz F.M., Wanders R.J. (2002). Carnitine biosynthesis in mammals. Biochem. J..

[92] Selhub J. (2002). Folate, vitamin B12 and vitamin B6 and one carbon metabolism. J. Nutr. Health Aging.

[93] Evangeliou A., Vlassopoulos D. (2003). Carnitine metabolism and deficit--when supplementation is necessary?. Curr. Pharm. Biotechnol..

[94] Kaida Y., Taguchi K., Fukami K. (2025). Carnitine Deficiency in Chronic Kidney Disease: Pathophysiology, Clinical Implications, and Therapeutic Perspectives. Nutrients.

[95] Kodentsova V.M., Risnik D.V., Kryukova E.V., Dariy S.G. (2024). L-carnitine: Food sources, adequate and clinically effective doses. Meditsinskiy Sov..

[96] De Grandis D., Minardi C. (2002). Acetyl-L-carnitine (levacecarnine) in the treatment of diabetic neuropathy—A long-term, randomised, double-blind, placebo-controlled study. Drugs R D.

[97] Packer L., Witt E.H., Tritschler H.J. (1995). alpha-Lipoic acid as a biological antioxidant. Free Radic. Biol. Med..

[98] Reed L.J. (1998). From lipoic acid to multi-enzyme complexes. Protein Sci..

[99] Reed L.J. (2001). A trail of research from lipoic acid to alpha-keto acid dehydrogenase complexes. J. Biol. Chem..

[100] Ziegler D., Low P.A., Litchy W.J., Boulton A.J., Vinik A.I., Freeman R., Samigullin R., Tritschler H., Munzel U., Maus J. (2011). Efficacy and safety of antioxidant treatment with α-lipoic acid over 4 years in diabetic polyneuropathy: The NATHAN 1 trial. Diabetes Care.

[101] Jacob S., Ruus P., Hermann R., Tritschler H.J., Maerker E., Renn W., Augustin H.J., Dietze G.J., Rett K. (1999). Oral administration of RAC-alpha-lipoic acid modulates insulin sensitivity in patients with type-2 diabetes mellitus: A placebo-controlled pilot trial. Free Radic. Biol. Med..

[102] Capece U., Moffa S., Improta I., Di Giuseppe G., Nista E.C., Cefalo C.M.A., Cinti F., Pontecorvi A., Gasbarrini A., Giaccari A. (2022). Alpha-Lipoic Acid and Glucose Metabolism: A Comprehensive Update on Biochemical and Therapeutic Features. Nutrients.

[103] Ziegler D., Nowak H., Kempler P., Vargha P., Low P.A. (2004). Treatment of symptomatic diabetic polyneuropathy with the antioxidant alpha-lipoic acid: A meta-analysis. Diabet. Med..

[104] Ametov A.S., Barinov A., Dyck P.J., Hermann R., Kozlova N., Litchy W.J., Low P.A., Nehrdich D., Novosadova M., O’Brien P.C. (2003). The sensory symptoms of diabetic polyneuropathy are improved with alpha-lipoic acid: The SYDNEY trial. Diabetes Care.

[105] Ziegler D., Hanefeld M., Ruhnau K.J., Hasche H., Lobisch M., Schütte K., Kerum G., Malessa R. (1999). Treatment of symptomatic diabetic polyneuropathy with the antioxidant alpha-lipoic acid: A 7-month multicenter randomized controlled trial (ALADIN III Study). ALADIN III Study Group. Alpha-Lipoic Acid in Diabetic Neuropathy. Diabetes Care.

[106] Ziegler D., Ametov A., Barinov A., Dyck P.J., Gurieva I., Low P.A., Munzel U., Yakhno N., Raz I., Novosadova M. (2006). Oral treatment with alpha-lipoic acid improves symptomatic diabetic polyneuropathy: The SYDNEY 2 trial. Diabetes Care.

[107] Zainal N.A., Abdul Rashid A.M., A Rauf A.L., Yusof Khan A.H.K., Wan Sulaiman W.A., Basri H. (2025). Evaluating the efficacy and tolerability of the oral combination of alpha lipoic acid and vitamin B complex preparation in carpal tunnel syndrome: A single center, randomized, double-blind, placebo-controlled trial. BMC Neurol..

[108] Mijnhout G.S., Kollen B.J., Alkhalaf A., Kleefstra N., Bilo H.J.G. (2012). Alpha Lipoic Acid for Symptomatic Peripheral Neuropathy in Patients with Diabetes: A Meta-Analysis of Randomized Controlled Trials. Int. J. Endocrinol..

[109] Yun S.M., Kim K.S., Lee H.J., Yoon H.J., Lee Y.S., Kim K.Y., Hyun D.W., Han S.W., Hur S.H., Lee C.W. (2004). The Relationship of Homocysteine, Vitamin B12, Vitamin B6 and Folate with Ischemic Heart Disease. Korean Circ. J..

[110] Gong Q., Wang B., Li L., Zhao G., Li C., Ma L., Yang H., Zhang X., An G., Guo C. (2026). Elevated methylmalonic acid, but not vitamin B12, predicts all-cause mortality in hyperlipidemic adults: A prospective cohort study. Front. Nutr..

[111] Sathienluckana T., Palapinyo S., Yotsombut K., Wanothayaroj E., Sithinamsuwan P., Suksomboon N. (2024). Expert consensus guidelines for community pharmacists in the management of diabetic peripheral neuropathy with a combination of neurotropic B vitamins. J. Pharm. Policy Pract..

[112] Lam J.R., Schneider J.L., Zhao W., Corley D.A. (2013). Proton Pump Inhibitor and Histamine 2 Receptor Antagonist Use and Vitamin B12 Deficiency. JAMA.

[113] van der Watt J.J., Harrison T.B., Benatar M., Heckmann J.M. (2011). Polyneuropathy, anti-tuberculosis treatment and the role of pyridoxine in the HIV/AIDS era: A systematic review. Int. J. Tuberc. Lung Dis..

[114] Shea B., Swinden M.V., Ghogomu E.T., Ortiz Z., Katchamart W., Rader T., Bombardier C., Wells G.A., Tugwell P. (2014). Folic acid and folinic acid for reducing side effects in patients receiving methotrexate for rheumatoid arthritis. J. Rheumatol..

[115] Romagnolo A., Merola A., Artusi C.A., Rizzone M.G., Zibetti M., Lopiano L. (2019). Levodopa-Induced Neuropathy: A Systematic Review. Mov. Disord. Clin. Pract..

[116] Rieck J., Halkin H., Almog S., Seligman H., Lubetsky A., Olchovsky D., Ezra D. (1999). Urinary loss of thiamine is increased by low doses of furosemide in healthy volunteers. J. Lab. Clin. Med..

[117] Mintzer S., Skidmore C.T., Sperling M.R. (2012). B-vitamin deficiency in patients treated with antiepileptic drugs. Epilepsy Behav..

[118] Sanz-Cuesta T., Escortell-Mayor E., Cura-Gonzalez I., Martin-Fernandez J., Riesgo-Fuertes R., Garrido-Elustondo S., Mariño-Suárez J.E., Álvarez-Villalba M., Gómez-Gascón T., González-García I. (2020). Oral versus intramuscular administration of vitamin B12 for vitamin B12 deficiency in primary care: A pragmatic, randomised, non-inferiority clinical trial (OB12). BMJ Open.

